# Serum placental growth factor as a biomarker of cerebrovascular changes in Alzheimer's disease

**DOI:** 10.1002/alz70856_100391

**Published:** 2025-12-25

**Authors:** Caroline Dallaire‐Théroux, Rosalie Cottez, Olivier Potvin, Mahsa Dadar, Vincent Emond, Cyntia Tremblay, Frederic Calon, Consortium d'Identification précoce de la Maladie d'Alzheimer ‐ Québec (CIMA‐Q)

**Affiliations:** ^1^ Université Laval / CHU de Québec, Quebec City, QC, Canada; ^2^ Université Laval, Quebec City, QC, Canada; ^3^ Quebec Heart and Lung Institute, Québec, QC, Canada; ^4^ Douglas Mental Health University Institute, Montréal, QC, Canada; ^5^ CHU de Quebec, Quebec, QC, Canada

## Abstract

**Background:**

While Alzheimer's disease (AD) biomarkers are now widely used, those focusing on vascular etiologies remain underdeveloped. Among candidates, angiogenesis markers appear particularly promising. Placental growth factor (PlGF) plays a key role in tissue vascularization and regulation of vascular permeability, in addition to being reactive to cerebral ischemia. Here, we aimed to establish the role of PIGF in AD by examining its association with selected MRI markers of cerebrovascular disease (CVD).

**Method:**

We measured serum concentrations of PIGF (U‐PLEX Human PIGF Assay, Meso Scale Discovery) in elderly individuals with subjective cognitive impairment (*n* = 114), mild cognitive impairment (*n* = 86), early AD (*n* = 29) and controls (*n* = 55) from the CIMA‐Q cohort. MRI CVD markers included regional white matter hyperintensity (WMH) volumes and microbleeds acquired through validated segmentation tools. PlGF levels were compared across groups using one‐way ANOVA, while associations with age, sex, vascular risk factors, cognition, and AD biomarkers were analyzed using Pearson/Spearman tests. Linear regression models adjusted for age, sex, and ApoE4 examined the link between PlGF and MRI CVD markers.

**Result:**

Serum PlGF was found to be higher in men than women (*p* = .02) and to increase with age (*p* = .003) but did not differ between clinical groups. Among vascular and metabolic parameters, PlGF levels correlated positively with waist size, weight and triglycerides levels (*p* <.005). There was also a trend between PlGF levels and diastolic, but not systolic, blood pressure (*p* = .05). Serum pTau217 was the only AD biomarker associated with PlGF (*p* <.05). We could not find any direct association between PlGF and cognition. PlGF levels correlated with total (r=0.19, *p* = .03) and deep cerebellar (r=0.18, *p* = .04) microbleeds, but not WMH. In regression models, the association between PlGF and microbleeds remain consistent in APOE4 carriers (b=2.1, *p* = .04) and in men (b=1.8, *p* = .03) only.

**Conclusion:**

Serum PlGF is a promising biomarker of CVD, especially cerebral microbleeds, in AD. Our results suggest that its role may specifically apply to certain subpopulations such as APOE4 carriers. In the era of anti‐amyloid therapies, understanding mechanisms linking PlGF to CVD is of utmost importance to evaluate its potential for safety and monitoring purposes.

